# Detection of Expressed *Otx* mRNA Isoforms in Sea Urchins by Mapping NGS Reads to Single-Gene/Transcript Sequences

**DOI:** 10.3390/biology15010072

**Published:** 2025-12-30

**Authors:** Mariia A. Maiorova, Yulia O. Kipryushina, Konstantin V. Yakovlev

**Affiliations:** Laboratory of Cytotechnology, A.V. Zhirmunsky National Scientific Center of Marine Biology, Far Eastern Branch, Russian Academy of Sciences, Palchevsky St. 17, 690041 Vladivostok, Russia; maiorovamariya@gmail.com (M.A.M.); yulia.kipryushina@gmail.com (Y.O.K.)

**Keywords:** sea urchin, *Otx*, isoforms, next-generation sequencing, single-gene mapping

## Abstract

The identification of mRNA isoforms in biological samples is crucial for studying tissue- and cell-specific isoform expression, activity of tissue-specific promoters, alternative splicing events, and alternative polyadenylation signals in genes. For single or several genes, expressed mRNA isoforms can be found using RT-PCR and RT-qPCR. Available transcriptome short-read archives deposited in GenBank or as laboratory data can be used to identify mRNA isoforms instead of or prior to wet analysis by other methods in eukaryotic organisms with annotated genomes. However, isoform expression analysis requires advanced bioinformatics skills and may be time-consuming. In addition, this analysis generates a large amount of unnecessary data. To detect mRNA isoforms encoded by one gene of interest, screening of expressed mRNAs in NGS data can be simplified by mapping NGS short reads to a single-gene or transcript sequence. Using single-gene/transcript mapping, we analyzed the expression of the *Otx* gene at the mRNA isoform level in some embryonic and adult tissue mRNA libraries of the sea urchin *Strongylocentrotus purpuratus* available in GenBank. The presence of expressed *Otx* mRNA isoforms was confirmed by RT-qPCR in the same tissues and at the same developmental stages of the closely related species *Strongylocentrotus intermedius*. We showed that single-gene/transcript mapping is a suitable approach for qualitative evaluation of the expression of mRNA isoforms and recognition of at least two expressed isoforms in the same biological sample.

## Simple Summary

Determination of mRNA isoform expression is important for studying tissue-specific gene regulation and the generation of protein diversity. We tested the potential of mapping to a single-gene/transcript for the qualitative examination of mRNA isoform expression produced from the same gene, including cases where several mRNA species are expressed in samples. Using single-gene/transcript mapping, we analyzed expression of the *Otx* mRNA isoforms in some mRNA libraries of the sea urchin *Strongylocentrotus purpuratus* available in GenBank. The presence of expressed *Otx* mRNA isoforms was verified by RT-qPCR in the same tissues and developmental stages of the closely related species *Strongylocentrotus intermedius*. We found that single-gene/transcript mapping can be easily applied to genes with a simple structure lacking overlapping exons and introns, such as *Otx* genes in sea urchins.

## 1. Introduction

Many eukaryotic genes have discontinuous structures consisting of exons and introns. Exons form the mRNA sequence during splicing, and introns are removed. Therefore, a single gene can encode different but related mRNA species, which are translated into different proteins called protein isoforms with diverse biological roles [1]. The appearance of different mRNA species is realized through alternative splicing, a process of exon–exon joining with different combinations. mRNA isoforms expressed from one gene can have different promoter regions if they differ in 5’UTR and transcription termination and polyadenylation sites if they have 3’UTR of different lengths [2,3].

To investigate the fine regulation of gene activity, splicing, and functions of different protein products of the same gene, it is necessary to know exactly which mRNA isoforms are expressed in the studied organism. Before the genomic era, mRNA isoforms were determined by sequencing cDNA libraries. Currently, predicted structures of genes and transcript sequences are available for many model organisms with annotated genomes. Information about expression of genes of interest (GOIs) can be found in databases, such as www.flybase.org for *Drosophila*, www.echinobase.org for several echinoderms, https://zfin.org for zebrafish, www.proteinatlas.org, www.gtexportal.org for human, and others. Although these databases often lack information on isoform expression, the predicted gene structures and encoded mRNA sequences facilitate the determination of specific isoform expression. For example, this can be easily performed by RT-PCR or RT-qPCR using primers that anneal isoform-specific sequences.

Another possible way to find expressed mRNA isoforms is the analysis of transcriptomic data, if they are available in the laboratory or deposited in GenBank by other investigators. Transcriptome analysis using next-generation sequencing (NGS) allows large-scale studies of RNA content. Transcriptomic analysis of mRNAs enables the identification of thousands of eukaryotic protein-coding RNA species and quantification of mRNA isoforms, known as differential isoform expression. Nevertheless, deep transcriptomic analysis at the isoform level requires advanced bioinformatic skills, such as the utilization of specialized software (e.g., DESeq, EdgeR) and expertise in scripting languages (e.g., Python, R).

Several investigators use short NGS reads to assemble and correct mRNA sequences of GOIs without performing full genomic and transcriptomic analyses, although this often goes unreported in publications. In this study, we examined how mapping of short Illumina reads against single-gene and mRNA sequences is applicable when it is necessary to detect the expression of GOI.

**Figure 1:**
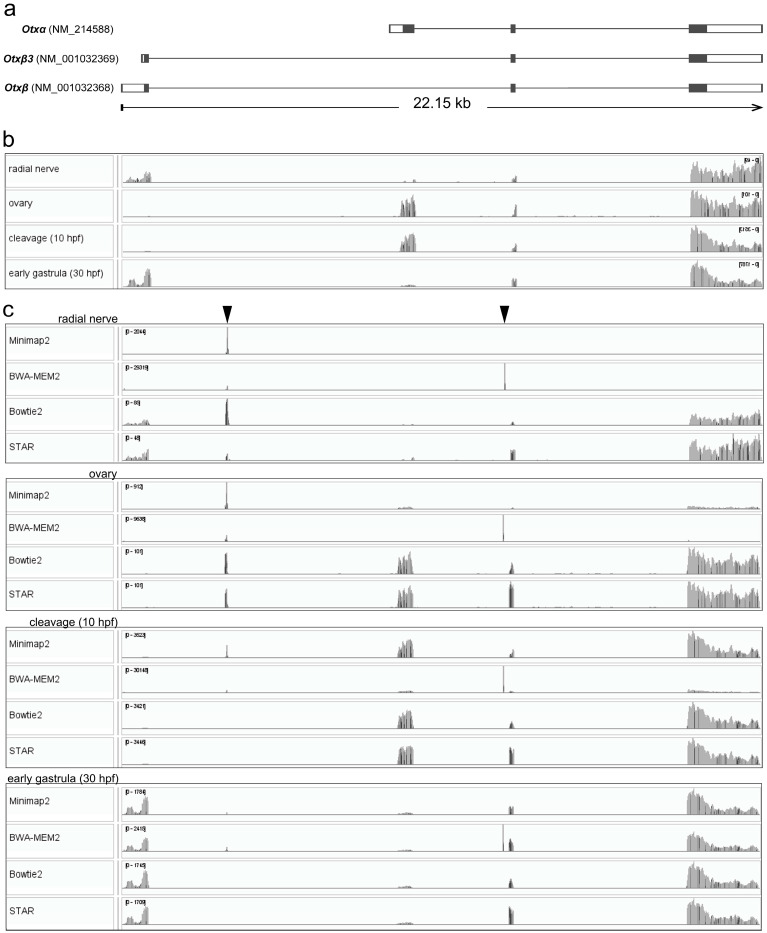
The *Otx* gene structure of *S. purpuratus* and mapping Illumina reads to the *Otx* gene sequence. (**a**) The *Otx* gene (GenBank Gene ID 373400) apparently encodes three mRNA species. The *Otxβ* and *Otxβ3* mRNAs differ by 5’UTR length and encode the same protein. Translated regions are highlighted in darker grey. (**b**) Coverage plot of raw reads from different tissues/developmental stages aligned to the *Otx* gene using Minimap2 after mapping to the *S. purpuratus* genome. single-gene sequence alignment. (**c**) Coverage plot of raw reads from different tissues/developmental stages aligned to the *Otx* gene after single-gene mapping using Minimap2, BWE-MEM2, Bowtie2, and STAR. Positions of non-specific peaks are marked by arrowheads. In (**b**,**c**), mapping coverages were aligned to the *Otx* gene structure (**a**).

Sea urchins are well-known models used in toxicology research and developmental biology. Populations of several sea urchin species are substantial components of coastal ecosystems. Edible species are important in fisheries and aquaculture. The first sea urchin genome of *Strongylocentrotus purpuratus* was published five years after the first release of the human genome [4,5]. Here, we used two sea urchin species of the genus *Strongylocentrotus* to test the potential of mapping to a single gene/transcript for the qualitative examination of mRNA isoform expression produced from the same gene, including cases where several mRNA species are expressed in samples. We demonstrated that by using this approach, we can detect the presence of *Otx* mRNA isoforms in early development and adult tissues of sea urchins. The *Otx* gene encodes homeobox orthodenticle-related proteins, homeobox-containing transcription factors. Published results and current GenBank data from two sea urchins, *S. purpuratus* (Gene ID: 373400) and *Hemicentrotus pulcherrimus* of the Strongylocentridae family, showed a conserved four-exon gene structure within this family [6,7]. In *S. purpuratus*, three *Otx* mRNA species, *Otxα*, *Otxβ*, and *Otxβ3*, encoding two Otx proteins, are likely to be generated from the *spOtx* gene through alternative transcription start site usage (Figure 1a). *spOtx* is expressed both maternally and zygotically during early sea urchin development and plays multiple roles in embryo patterning [8,9,10,11,12,13,14,15,16,17,18]. *Otxα* is expressed maternally and zygotically from oogenesis to early gastrula, whereas *Otxβ* is expressed from the blastula stage. These mRNA species exhibit two distinct temporal and spatial expression patterns [7,17]. At later stages, *Otx* expression was found in photoreceptor cell types of adult rudiment of *Paracentrotus lividus* plutei [13]. In addition, *Otx* was detected in larval nervous system of *Holopneustes purpurascens* [12].

Known expression of *Otx* at the mRNA isoform level in early sea urchin development allows the use of *Otx* as an appropriate model for the detection of gene expression by mapping to single-gene/transcript sequences. In this paper, we employed single-gene/transcript mapping to detect the *Otx* mRNA species in embryonic and adult tissue mRNA libraries of *S. purpuratus* available in GenBank. Further, we confirm the presence of the mRNA isoforms by RT-qPCR in the same tissues and developmental stages of the closely related species *Strongylocentrotus intermedius*. Next, we discuss the pros and cons of single-gene/transcript mapping for the detection of expressed mRNA isoforms and how this approach is feasible for researchers with basic NGS analysis skills.

## 2. Materials and Methods

### 2.1. NGS Data Analysis

*S. purpuratus* SRAs used for mapping are available in GenBank (BioProject PRJNA81157). We used SRR532046 from the radial nerve, SRR531958 from the ovaries, SRR531949 from the cleavage stage (10 h post fertilization (hpf)), and SRR532074 from early gastrula (30 hpf) [19,20]. The libraries were passed FastQC (Version 0.12.1) quality control showed high per-base and per-sequence quality scores.

Illumina mRNA libraries were prepared from total RNA samples from unfertilized *S. intermedius* eggs. Raw 2 × 100 and 2 × 150 pair-end reads were generated using the Illumina NovaSeq 6000 platform, performed by Macrogen (Seoul, Republic of Korea) and Novogene (Beijing, China), respectively. The libraries were passed FastQC quality control showed high per-base and per-sequence quality scores. SRAs were submitted to GenBank (BioProject PRJNA686841). *Otxα* sequence was generated from transcript fragments found in the previously published egg transcriptome [21]. Sequence of the *Otxβ*-specific exon absent in eggs was obtained by alignment of the *S. intermedius* genomic reads (PRJNA391452) [22] to the *S. purpuratus Otx* gene using Minimap2 (Version 2.28) [23]. This program was also used for mapping of transcriptomic reads against the *S. purpuratus* genome. Transcriptomic reads were mapped to the *Otx* gene using Minimap2, BWA-MEM2 (Version 2.3), Bowtie2 (Version 2.5.4), and STAR (In Galaxy, this program is named RNA STAR. Version 2.7.11b) [23,24,25,26,27]. Mappings against the *Otx* transcripts were performed by Minimap2 and STAR. Then, reads were sorted by mapping status using BAMTools (Version 2.5.2) [28]. Mapping and sorting of reads were performed in the web-based Galaxy platform (https://usegalaxy.org) with default conditions [29]. Default conditions for each aligner are given in Supplementary Materials (Tables S1–S4). Alignments were visualized using IGV v.2.16.2 [30] and Unipro UGENE v.51.0 [31]. A generalized guide for using Galaxy web platform and visualization is provided in Supplementary Materials (File S1).

### 2.2. Animals and Sample Preparations

Adult sea urchins *S. intermedius* more than 4 cm in diameter were collected in different locations of the Peter the Great Bay (Sea of Japan) from 2–5 m depth throughout the year, kept in aquaria with aerated seawater at 15–17 °C, and fed with algae (*Ulva fenestrata* and *Saccharina japonica*) at least twice a week. To obtain gametes, we used naturally matured animals collected in April–May and August–September or maturation was induced by keeping sea urchins in aquaria for at least two months with unlimited food. Spawning was stimulated by injecting sea urchins with 0.5 M KCl. Eggs from two or three females were mixed, washed several times with filtered seawater and fertilized. Embryos were raised in climate chamber at 15 °C and collected at 6 hpf (32-cell stage) and 25 hpf (early gastrula). Embryos were frozen and stored at −80 °C. Dissected radial nerves of two–three animals and ovaries at mature stage (one animal) were washed with filtered seawater and Ca^2+^- and Mg^2+^-free salt solution (CMFSS, 12 mM HEPES, pH 7.6–7.8, 385 mM NaCl, 10 mM KCl, 21 mM Na_2_SO_4_, 17 mM glucose) and then frozen at −80 °C.

### 2.3. RNA Isolation

For NGS analysis, total RNA was extracted from *S. intermedius* eggs by using PureLink Mini kit (Thermo Fisher Scientific, Waltham, MA, USA). For RT-qPCR, RNA samples were isolated from frozen samples by using TRIzol Reagent (Thermo Fisher Scientific, Waltham, MA, USA) according to the manufacturer’s protocol with one exception. Some pigment content irreversibly bound to RNA in radial nerve samples after alcohol addition, which resulted in pink colored RNA. These pigments inhibited enzymatic reactions, most likely at cDNA synthesis stage. In order to prevent co-precipitation of undesirable pigment content, RNA from radial nerves was precipitated by addition of LiCl to a final concentration of 3 M and then washed with ethanol.

### 2.4. RT-qPCR

The first strand of cDNA was synthesized by RNAscribe RT (Biolabmix, Novosibirsk, Russia) according to the manufacturer’s protocol. Briefly, the 20 µL reaction contained 600 ng of total RNA and 1 µL of primer mix (mixture of random hexanucleotide and oligo(d)T_16_ primers) was incubated for 10 min at 25 °C and then for 30 at 55 °C. Reaction was stopped by incubation at 85 °C for 5 min and then cDNA was diluted three times to final volume of 60 μL. RT-qPCR was conducted on the CFX96 Touch Real-Time PCR Detection System (Bio-Rad, Hercules, CA, USA) using qPCRmix-HS SYBR master mix (Evrogen, Moscow, Russia). The 25 µL reaction mixture contained 2 μL of diluted template cDNA and 0.25 μM of each primer (Table 1) with the following temperature program: 94 °C for 30 s, 40 cycles of 94 °C for 15 s, 56 °C for 30 s, and 72 °C for 15 s. Each sample was analyzed in at least three technical replicates. The relative expression levels were examined by the ΔΔCt method using *Ubb* as a reference gene [21,32,33,34,35]. Plots were created using the ggplot2 package in R Statistical Software version 4.1.0.

## 3. Results

### 3.1. Application and Visualization of Single-Gene/Transcript Mapping

We used *S. purpuratus* raw reads available in GenBank from ovaries, cleavage, and early gastrula [19,20]. As *Otx* is expressed in the larval nervous system, we examined the radial nerve transcriptome of adult *S. purpuratus*. All transcriptomes were aligned to the *S. purpuratus* genome (GenBank, GCA_000002235.4) using Minimap2, which showed *Otx* expression in all cases (Figure 1b). Clear read coverage patterns allowed the recognition of expressed mRNA isoforms. The *Otxβ* isoform is expressed in the radial nerve. Also, two minor peaks were detectable in the *Otxα*-specific exon, although the exon coverage was partial, i.e., it had discontinuity. Based on these results, we concluded that *Otxα* is not expressed in the radial nerves. *Otxα* is expressed in the ovaries and at the cleavage stage. In the early gastrula, read coverage was detected for both *Otxα*- and *Otxβ*-specific exons, demonstrating expression of both isoforms. While *Otxβ3* expression is absent in ovaries and at the cleavage stage, its expression in the radial nerve and the early gastrula stage remains undetermined. This uncertainty arises because the *Otxβ3* transcript start site lies within the *Otxβ*-specific exon, which prevents isoform-specific mapping. Employment of a short reference sequence for mapping may lead to increased non-specific coverage depth. To test this statement, we mapped reads to the *Otx* gene sequence using Minimap2, BWA-MEM2, Bowtie2, and STAR (Figure 1c). Unlike whole-genome alignment, mapping to the *Otx* gene sequence alone demonstrated a reduced specificity. Many coverage profiles of the alignments to the single-gene reference contained one or two narrow nonspecific peaks within the introns. These peaks were generated in repetitive sequences with approximate lengths of 20 and 76 nucleotides (Figure 1c). In the radial nerve, Minimap2 produced only one non-specific coverage peak of the repetitive intronic sequence. Two non-specific peaks were observed in the ovaries after BWA-MEM2 mapping. Indeed, the exon regions contained mapped reads after Minimap2 and BWA-MEM2 mapping. Exon coverage by raw reads was invisible in the general view of the plots, but could be found in the detailed view of the exon regions (Figure S1). Evidently, specific coverage in these plots is invisible due to automatic scaling of the plots by visualizers based on high non-specific coverage in repetitive sequences. Other alignments contained both non-specific and exon-specific coverage, which results in increased accuracy of the alignment. Only Bowtie2 and STAR showed superior mapping without non-specific peaks at the cleavage and early gastrula stages (Figure 1c). We concluded that among the tested programs, STAR and Bowtie2 showed the best mapping specificity, whereas other mappers revealed less accurate alignments (Table 2).

**Figure 2:**
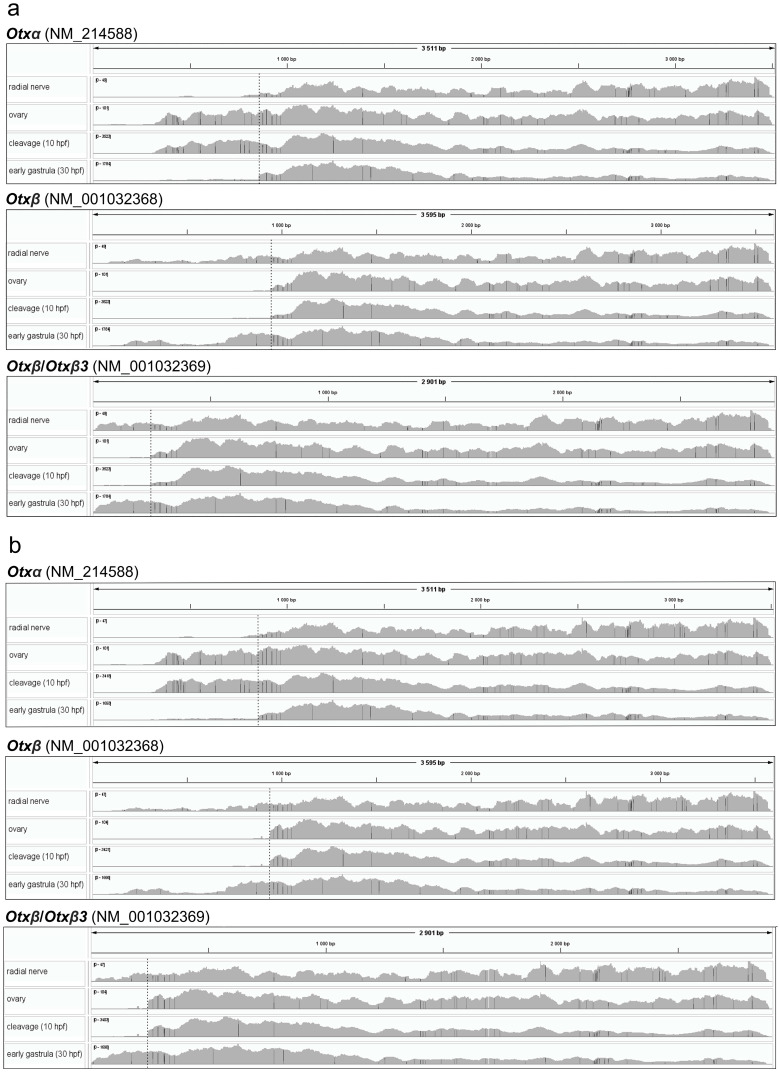
Coverage plots of raw reads from different tissues/developmental stages aligned to each *Otx* transcript sequence. (**a**) Coverage plot generated using Minimap2. (**b**) Coverage plot generated using STAR. Frame lengths correspond to transcript sequence lengths. Dashed lines mark the exon1-exon2 junctions.

Single transcript mapping was performed for each *Otx* mRNA isoform sequence (GenBank Gene ID 373400). We tested Minimap2 and STAR as mapping programs that showed less and more accuracy in alignment to the *Otx* gene sequence, respectively. Both mappers generated similar coverage plots and were discussed together (Figure 2). Since *Otx* isoforms differ in their first exons but share identical downstream sequences, we determined expression of the isoforms by coverage of the first exon sequences (Figure 2). According to the mapping data, *Otxα* is expressed in the ovaries and at the cleavage and early gastrula stages. The first exon of *Otxα* showed no 5’-end coverage, with aligned reads beginning at the same downstream position across the ovaries, cleavage, and gastrula stages. This indicates that the transcription start site (TSS) for *Otxα* is located downstream of its position, as given in the *Otx* gene model. The downstream location of the TSS is supported by the experimental determination of the 5’UTR sequence [7]. Furthermore, an ortholog of *Otxα*, *HpOtxE*, and closely related species *H. pulcherrimus* has a similar position of TSS [6]. The 5’UTR sequence of assembled de novo *Otxα* of *S. intermedius* also confirmed the downstream TSS location. Therefore, the *S. purpuratus Otx* gene model should be revised accordingly. *Otxβ* is expressed at the early gastrula stage and in the radial nerve. In the latter case, we observed two coverage zones within the *Otxα*-specific exon, similar to whole-genome alignment. This fragmented coverage provides only limited evidence for *Otxα* expression. In early gastrula, both *Otxα* and *Otxβ* were detected by both single-gene and transcript mapping (Figure 1b,c and Figure 2). The sharp increase in *Otxα* coverage at the junction between its first specific exon and the common *Otx* sequence suggests differential expression levels between the isoforms. Potential *Otxβ3* expression could be masked by the *Otxβ* coverage profile in both single-gene and whole-genome alignment approaches.

**Figure 3:**
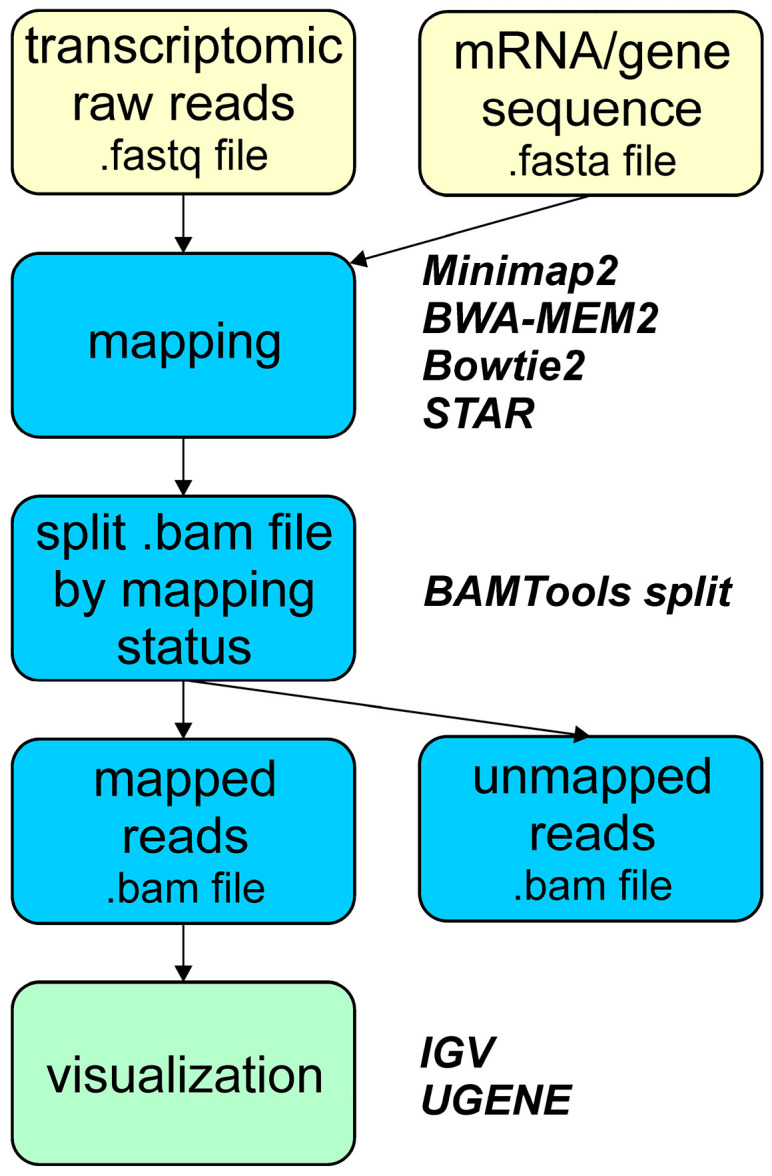
Overview of the pipeline used for raw read mapping to a single mRNA/gene sequence to qualitatively estimate expression of the *S. purpuratus Otx* gene and its mRNA isoforms.

The implemented mapping pipeline for alignment of raw reads to single-gene/transcript sequences is given in Figure 3. This includes all used mapping transcriptomic reads, the read filtration step, and visualization. Read filtration separates mapped from unmapped reads. While this step may seem, the resulting files containing only mapped reads are smaller in size, facilitating more efficient subsequent analysis and visualization on desktops and laptops.

### 3.2. RT-qPCR Analysis of Otx Isoforms Expression in S. intermedius Samples

**Figure 4:**
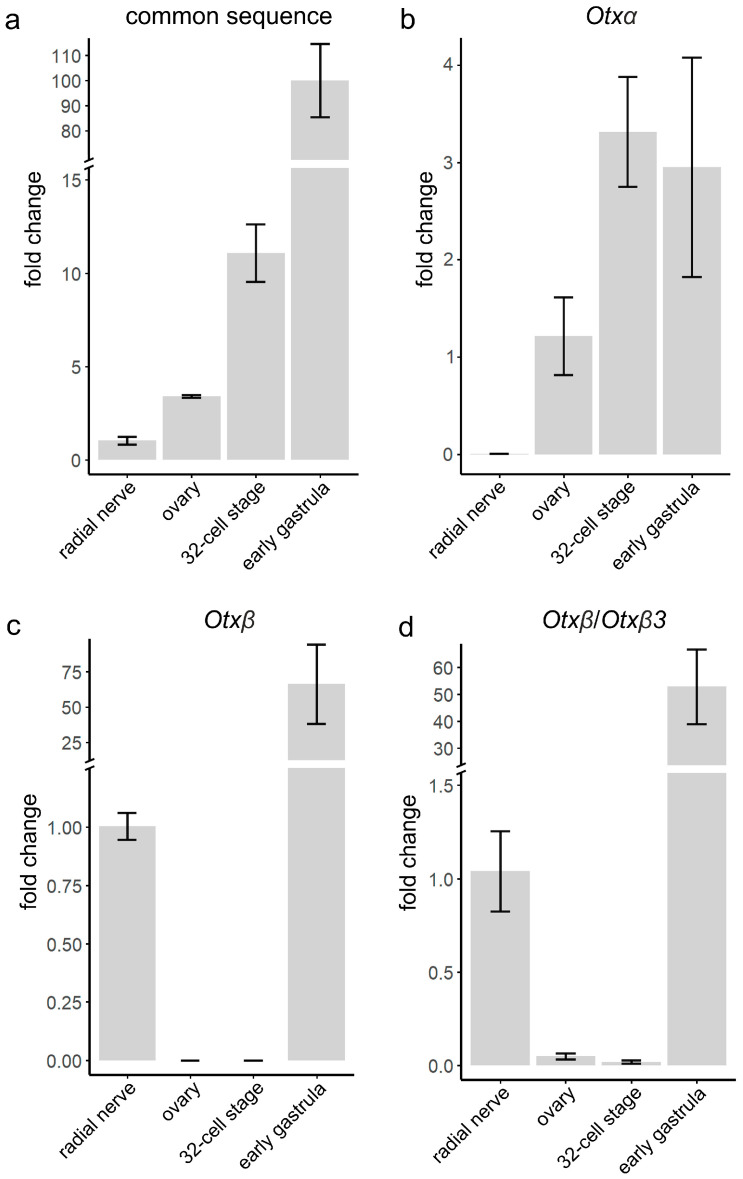
RT-qPCR analysis of relative expression levels of *Otx* mRNA species in *S. intermedius* adult and embryonic samples. (**a**) Total expression of all isoforms was analyzed using oligos annealing within the common sequence. Expressions of *Otxα* (**b**) and *Otxβ* (**c**) were analyzed using sequence-specific oligos. (**d**) Expression of *Otxβ*/*Otxβ3* common sequence. Expression levels were calculated related to the radial nerve (**a**,**c**,**d**) and ovaries (**b**). Values are shown as mean ± SEM. Three independent biological replicates were analyzed for each tissue and developmental stage.

To confirm and extend the *Otx* isoform expression results based on read mapping, we performed RT-qPCR analysis in *S. intermedius*, which inhabits the coastal seawater of the Sea of Japan. Due to the restricted distribution of most sea urchin species, we chose *S. intermedius* as the closest related species to *S. purpuratus* among sea urchins available to us. Both species are phylogenetically close, should have the same *Otx* gene structure, and very similar development and anatomy. Based on these facts, we suspect that the expression pattern of the *Otx* gene in these species would be similar. We examined expression in the same developmental stages and adult tissues analyzed by single-gene/transcript mapping in *S. purpuratus*. First, we analyzed total *Otx* expression using oligos annealing to the common sequence (Figure 4a). *Otx* expression was detected in all samples with the lowest levels in the radial nerve (1) and the highest in the early gastrula (100-fold increase). Next, we used oligos annealing to the sequences of the first isoform-specific exons. *Otxα* is expressed in the ovaries, at the 32-cell stage, and in the early gastrula. The lowest level was detected for ovaries (1.2) in comparison with 32-cell (3.3) and early gastrula (2.95) stages (Figure 4b). *Otxβ* is expressed in the radial nerve and early gastrula. In the early gastrula, *Otxβ* expression level was significantly higher (100-fold increase) than in the radial nerve (Figure 4c). *Otxβ3* expression could not be estimated because the sequence of the *Otxβ3* first exon is a part of the longer *Otxβ*-specific exon. Therefore, RT-qPCR allows us to analyze the total expression of both isoforms (Figure 4d). As illustrated in Figure 4b–d, the expression levels of *Otxα* in radial nerve and *Otxβ* in ovaries and 32-cell embryos were very low, which we considered as background levels. These faint signals might be a result of little DNA contamination or basal isoform expression. The highest value among faint signals was for *Otxβ*/*Otxβ3* in the ovaries, which was 5% of the expression level in the radial nerve (Figure 4d). In conclusion, RT-qPCR analysis confirms the results of *Otx* expression on mRNA isoform level obtained by single-gene/transcript mapping.

## 4. Discussion

Single-gene/transcript mapping requires basic NGS analysis skills, such as basic processing of the NGS read libraries, mapping, and visualization of mappings. This approach can be used to quickly obtain qualitative expression results for RNA isoforms, provided transcriptome raw reads are available. Although mapping to a single gene may be less specific than whole-genome mapping, additional mapping to transcript sequences enables accurate determination of expressed isoforms. Non-specific signals in the coverage profile from single-gene mapping can be disregarded because transcribed exons are visible, allowing for the definition of expressed RNA isoforms. While the runtimes for whole-genome and single-gene mappings are comparable, the results files from mapping to a single gene are smaller. This reduced file size is far more convenient for analysis on desktops and laptops.

Several RT-qPCR variations are often used to determine mRNA isoform expression. In these cases, expression of particular isoforms is estimated by measuring isoform-specific exons or detecting exon–exon junctions [36,37,38]. RT-qPCR allowed us to confirm expression of the *Otx* isoforms identified by single-gene/transcript mapping in different *S. intermedius* samples. However, RT-qPCR did not allow the verification of transcription start and end sites. Single-gene/transcript mapping revealed that *Otxα* possesses TSS downstream of the predicted location in the *Otx* gene model, which matches the TSS experimentally determined earlier [6,7]. We consider that single-gene/transcript mapping is appropriate for qualitative isoform expression analysis and predicted sequence verification. At the same time, the single-gene mapping method is not suitable for estimating gene expression levels for several reasons: it does not account for background expression levels (most expression quantification methods count all reads and normalize for library size), and some reads may map to multiple transcripts, leading to inflated expression estimates (this issue is often addressed using Expectation–Maximization algorithms).

Time required for the analysis by single-gene/transcript mapping was less than we spent on RT-qPCR. The mapping against the *Otx* gene and its transcripts using the established scheme took approximately a week. This included downloading available read libraries, checking read quality, mapping using multiple mappers, and visualizing the resulting data. RT-qPCR took us approximately two months. This included obtaining biological material and RNA isolation, primer design and validation, cDNA synthesis, expression analysis in multiple biological replicates, and calculating expression levels and plotting generation. This suggests that the mapping is a faster way to identify expressed isoforms compared to RT-qPCR. However, if quantitative expression results are required, RT-qPCR or differential transcriptome expression analysis at the isoform level should be done.

Single-gene/transcript mapping has some limitations. First, this approach can be employed in cases where an annotated genome is available. Transcriptome data without a known exon-intron structure of the gene do not allow mapping and examining coverage of exon sequences by raw reads. The exon-intron structure of the gene and the expression of different mRNA isoforms can be indirectly confirmed using the same approach we used. We first mapped reads to the *Otx* sequence of *S. purpuratus*, a species with an annotated genome, and confirmed the presence of two isoforms in the closely related species *S. intermedius*, which lacks an annotated genome. However, this approach may not be successful for other genes in other animals.

Next, not all mapping programs are convenient for single-gene mapping. In our analysis, STAR and Bowtie2 were appropriate for gene mapping, whereas BWA-MEM2 and Minimap2 showed significant non-specific coverage of repetitive sequences by reads in introns. This may complicate analysis of the *Otx* mRNA isoform expression, as non-specific coverage is significantly higher than coverage of exons, which may be invisible in the general view of coverage plots. In these cases, the coverage of exons can be estimated by viewing the exon regions. However, the best option would be the use of mapping programs that provide more accurate coverage depth. As we showed, mapping non-specificity is more pronounced at low *Otx* expression. According to our results, high non-specific coverage peaks appeared in the radial nerve and ovaries. In these tissues, the highest non-specificity correlates with the lowest *Otx* expression estimated by RT-qPCR. In the early gastrula, RT-qPCR analysis showed the highest *Otx* expression, and nonspecific signals were low compared to other samples or invisible, depending on the chosen mapping program. In contrast, Minimap2 showed successful alignment of reads against a single transcript sequence, similar to STAR. However, this fact does not exclude the emergence of non-specific alignment when Minimap2 is used for mapping reads to other genes. Thus, our results show that the appearance and level of non-specific alignments in repetitive sequences depend on the chosen mapping program. Also, non-specific alignment is more pronounced at the lower *Otx* expression levels, while at the high *Otx* expression levels, non-specificity is reduced or invisible. Non-specific mapping, particularly on repetitive sequences, may be defined by visually examining the sequences. If a strong signal appears in intronic regions, it is necessary to examine the sequence itself. If it consists of short repeats, the mapping in this region is most likely non-specific. Then, we suggest performing mapping using several programs to compare the results and select mapping coverage plots without or with minimal non-specificity.

Single-gene/transcript mapping against the *Otx* gene and its transcripts revealed the potential masking of short UTRs by longer ones. In this case, the mapping did not allow differentiation of the presence of the *Otxβ3* isoform in the radial nerve and early gastrula. Expression of the *Otxβ* isoform with the longer 5’UTR leads to the generation of the coverage profiles, which overlay the start of the 5’UTR of *Otxβ3*. Therefore, single-gene/transcript mapping can detect the longest UTR among isoforms that differ by their UTR lengths. Using methods reliable to estimate the UTR length of each isoform, such as RACE or long-read sequencing, can solve this problem.

Single-gene/transcript mapping is most easily applied to genes with a simple structure lacking overlapping exons and introns, such as *Otx* genes in sea urchins. The complex structure of genes containing numerous exons and encoding multiple mRNA isoforms generated by alternative splicing can significantly complicate analysis. The kinesin light chain gene of *S. purpuratus* serves as an illustrative example (Figure S2, Table S5). In this case, it is informative to perform mapping against individual transcript sequences. The resulting alignments should be carefully examined for reads spanning exon–exon junctions. If a specific junction is covered by reads, the analyzed transcript is likely to be expressed (Figures S3–S5). Contrariwise, if the junction is not covered by any reads or if the coverage is low, this may indicate the absence of the corresponding transcript isoform. Furthermore, gaps in the coverage profile, which indicate the lack of expression for part or an entire exon, should be taken into account (Figures S6–S8). Based on this analysis, expressed isoforms of kinesin light chain can be determined. However, additional verification should be made, for example, by RT-PCR or RT-qPCR. However, this analysis will also be a challenging task due to the expression of several isoforms and overlapping exons. Alternatively, long-read sequencing may be used to detect mRNA isoforms [39]. However, the cost of long-read sequencing is too high for analyzing the expression of only one or a few genes.

## 5. Conclusions

In summary, we showed that the expression of sea urchin *Otx* mRNA isoforms can be detected by single-gene/transcript mapping. According to our results, single-gene/transcript mapping is a rapid process that can be employed instead of RT-PCR in cases of available annotated genomes and transcriptomic libraries. The mapping results can be confirmed and extended by quantitative analysis, such as RT-qPCR. The sea urchin *Otx* gene has a simple gene structure consisting of four non-overlapping exons, which allows for easy recognition of exon-specific expression. However, single-gene/transcript mapping did not allow us to differentiate the *Otx* isoforms that differ by their 5’UTRs. In this case, this approach could only detect the longest 5’UTR. We hypothesize that single-gene/transcript mapping can also be employed for other genes with simple gene structures in any eukaryotic organism with known genome and sequenced transcriptomes. More complex genes with numerous and overlapping exons can complicate the interpretation of mapping results. Therefore, we do not recommend using this approach alone without confirmation by other methods, such as RT-qPCR or long-read sequencing. Despite potential drawbacks, such as non-specific mapping to a single gene and limited software compatibility, the combined use of single-gene and transcript mapping in conjunction with testing different aligners yields relevant and highly informative results that identify the expressed mRNA isoforms.

## Figures and Tables

**Table 1:** Sequences of oligos used for RT-qPCR.

Sequence (GenBank Accession Number)	Oligos	Product Size, bp
Common sequence	F: TGTTAAAATGAACCACCACCAATC R: AGCAACATTCGATGATAATCGTTC	146
*Otxα* (PV009158)	F: TCAACAGCGTTATCAGCTGGAC R: TTGATTTCACGACTAGCAAGATCAG	130
*Otxβ* (PV009157)	F: CGAATATGTGCGCTTAACGAGT R: TGCGTGTCAAATTACAAAGCAAG	180
*Otxβ* (PV009157) and *Otxβ3* (putative) common sequence	F: CTGGATCATTCTGCCTTGACAG R: TGTTCTGAAGGTGGTGGTGATG	106

**Table 2:** Specificity and exon coverage after mapping against the *Otx* gene sequence.

Tissue/Stage	Minimap2		BWA-MEM2		Bowtie2		STAR	
	Exon Coverage	Non- Specificity	Exon Coverage	Non- Specificity	Exon Coverage	Non- Specificity	Exon Coverage	Non- Specificity
**Radial nerve**	–	+	–	+	+	+	+	+
**Ovary**	+	+	–	+	+	+	+	+
**Cleavage (10 hpf**)	+	+	+	+	+	–	+	–
**Early gastrula (30 hpf)**	+	+	+	+	+	–	+	–

Exon coverages were estimated from general view of the plots. “+” in “non-specificity” columns indicates spurious peaks. Alignments without non-specific mapping were identified as best (highlighted with bold borders).

## Data Availability

All SRAs and sequences cited in the text are available in GenBank.

## Supplementary Materials

The following supporting information can be downloaded at: https://www.mdpi.com/article/10.3390/biology15010072/s1. Table S1: Default parameters of the alignment of cleavage stage (10 hpf) reads against the *Otx* gene using Minimap2; Table S2: Default parameters of the alignment of cleavage stage (10 hpf) reads against the *Otx* gene using BWA-MEM2; Table S3: Default parameters of the alignment of cleavage stage (10 hpf) reads against the *Otx* gene using Bowtie2; Table S4: Default parameters of the alignment of cleavage stage (10 hpf) reads against the *Otx* gene using STAR; Figure S1: Coverage plot of the *Otx* gene third (last) exon by raw reads of the radial nerve. 5’ end of the exon is marked by a dashed line; Table S5: Expression analysis of six mRNA isoforms of the kinesin light chain protein gene (LOC373373) by single transcript mapping in radial nerve (SRR532046); Figure S2: Structure of kinesin light chain gene (LOC373373). Analyzed isoforms are marked by letters. N—non-expressed isoforms, E—expressed isoforms. Region encoding alternatively spliced exons and alternative polyadenylation signals for the XM sequences is between the arrows. The number of exons that are mentioned in the next figures is listed below for each transcript sequence; Figure S3: XM_030998283. transcript. (a) Coverage profile for the entire sequence. (b) Variable region; Figure S4: XM_030998288 transcript. (a) Coverage profile for the entire sequence. (b) Variable region; Figure S5: XM_011676658 transcript. (a) Coverage profile for the entire sequence. This transcript has shorter 3’UTR, and the coverage profile does not allow for checking this sequence by mapping. (b) Variable region. Gaps in read mapping (black lines) show alternative splicing of short exon; Figure S6: XM_030998282 transcript. (a) Coverage profile for the entire sequence. (b) Variable region. Coverage of the exon 12 3’ end is absent. Therefore, exon 13 is not expressed. The ends of the exon 12 and exon 13 are marked in both images; Figure S7: XM_030998279 transcript. (a) Coverage profile for the entire sequence. (b) Variable region. Exon 13 is not expressed, reads almost non-overlap exon14-exon15 junction. Exons 13 and 14 are marked in both images; Figure S8: XM_030998284 transcript. (a) Coverage profile for the entire sequence. (b) Variable region. Reads almost non-overlap exon14-exon15 junction. Exon 14 is marked in both images. File S1: A generalized guide was compiled for using the Galaxy web platform.
