# Supplementary material for: Detection of Expressed Otx mRNA Isoforms in Sea Urchins by Mapping NGS Reads to Single-Gene/Transcript Sequences
Source: Biology (Basel). 2025 Dec 30;15(1):72. doi: 10.3390/biology15010072 (PMC12784935; doi:10.3390/biology15010072)
Supplement: Supplementary file 1 [file biology-15-00072-s001.zip › File S1.pdf]

A generalized guide was compiled for using the Galaxy web platform

Analysis in Galaxy

- Upload own libraries as .fastq files or upload libraries inserting their SRR IDs from NCBI using ‘Faster Download and Extract Reads in FASTQ’ tool.
- Upload separate .fasta files with gene and transcript files. Gene and mRNA sequences can be found in GenBank, Ensembl, and in other databases.
- Check quality of the library reads using FastQC without uploading Contaminant and Adapter lists. Focus primarily on passing by per base and per sequence quality scores.

Mapping

- Choose mapping program.

Minimap2

- Parameter ‘Will you select a reference genome from your history or use a built-in index?’ → ‘Use a genome from history and build index’ → an appropriate .fasta file with gene or mRNA sequence.
- Parameter ‘Single or Paired-end reads’ → choose appropriate fastq file(s) with reads.
- Run.

BWA-MEM2

- Parameter ‘Will you select a reference genome from your history or use a built-in index?’ → ‘Use a reference from history and build index, if necessary’ → an appropriate .fasta file with gene or mRNA sequence.
- Parameter ‘Single or Paired-end reads’ → choose appropriate fastq file(s) with reads.

‘paired’ if Forward (F) and Reverse (R) reads are in separate file.

‘paired collection’ if F and R reads are in collection.

‘paired interleaved’ if F and R reads have been combined into a single file.

- Run

Bowtie2

- Parameter ‘Is this single or paired library?’ → choose appropriate fastq file(s) with reads.
- Parameter ‘Will you select a reference genome from your history or use a built-in index?’ → ‘Use a genome from the history and build index’ → an appropriate .fasta file with gene or mRNA sequence.
- Run

RNA STAR

- Parameter ‘Single-end or paired-end reads’ → choose appropriate fastq file(s) with reads.

‘paired-end (as individual datasets)’ if Forward (F) and Reverse (R) reads are in separate file.

‘paired collection’, if F and R reads are in collection.

- Parameter ‘Custom or built-in reference genome’ → ‘Use reference genome from history and create temporary index’ → an appropriate .fasta file.

Parameter ‘Length of the SA pre-indexing string’ – default option is 14, but smaller value may be selected.

- Run

Filtering mapped reads

- Run ‘Split BAM by reads mapping status’ with generated .bam files.
- Download .bam and .bai (bam index) files of mapped reads on personal computer or laptop.

Analysis on laptop

Visualization

UGENE

Requirements: .bam file.

- Open .bam file, UGENE automatically generates and opens .ugene file. It is necessary to click on EXPORT reference sequence in pop-up window, when file is opening.

IGV (2.16.2)

Requirements: .bam file, .bai file, .fasta file with reference sequence.

- Choose an appropriate .fasta file used for mapping as Genome.
- Choose an appropriate .bam file in ‘File/Load from file’ menu. **.bai file is required to open the .bam file.**.
