# Supplementary material for: Detection of Expressed Otx mRNA Isoforms in Sea Urchins by Mapping NGS Reads to Single-Gene/Transcript Sequences
Source: Biology (Basel). 2025 Dec 30;15(1):72. doi: 10.3390/biology15010072 (PMC12784935; doi:10.3390/biology15010072)
Supplement: Supplementary file 1 [file biology-15-00072-s001.zip › Supplementary Materials_R1.pdf]

Table S1. Default parameters of the alignment of cleavage stage (10 hpf) reads against the *Otx* gene using Minimap2.

Input Parameter	Value
Will you select a reference genome from your history or use a built-in index?	history
Use the following dataset as the reference sequence	NM_001032368_genomic.fa
Single or Paired-end reads	paired_collection
Select a paired collection	SRR531949
Presets for PE reads alignment	Short reads without splicing (-k21 -w11 --sr -F800 -A2 -B8 -O12,32 -E2,1 -r50 -p.5 -N20 -f1000,5000 -n2 -m20 -s40 -g200 -2K50m --heap-sort=yes --secondary=no) (sr)
indexing_options	
Use homopolymer-compressed k-mer ?	false
K-mer size	Not available.
Minimizer window size	Not available.
Split index for every N input gigabases	Not available.
mapping_options	
Retain at most INT secondary alignments	Not available.
Max fragment length for PE alignment	Not available.
Filter out top FLOAT fraction of repetitive minimizers	Not available.
Specify an interval of k-mer occurrences	
Force minimap2 to always use k-mers occuring this many times or fewer	Not available.
Discard a query minimizer	0.01
Stop chain enlongation if there are no minimizers in INT-bp	Not available.

Input Parameter	Value
Bandwidth used in chaining and DP-based alignment	Not available.
Minimal number of minimizers on a chain	Not available.
Minimal chaining score (matching bases minus log gap penalty)	Not available.
Maximum seed skips during chaining	Not available.
Maximum number of partial chains checked during chaining	Not available.
Skip self and dual mappings (for the all-vs-all mode)	false
Min secondary-to-primary score ratio	Not available.
Fine control the removal of redundant hits	Not available.
alignment_options	
Customize spliced alignment mode?	preset
Score for a sequence match	Not available.
Penalty for a mismatch	Not available.
Gap open penalties for deletions	Not available.
Gap open penalties for insertions	Not available.
Gap extension penalties; a gap of size k cost '-O + -E*k'. If two numbers are specified, the first is the penalty of extending a deletion and the second for extending an insertion	Not available.
Gap extension penalty for extending an insertion; if left empty uses the value specified for Gap extension penalties above	Not available.

Input Parameter	Value
Z-drop threshold for truncating an alignment	Not available.
Z-drop threshold for reverse-complementing the query	Not available.
Minimal peak DP alignment score	Not available.
Filter seeds towards the ends of chains before performing base-level alignment?	true
io_options	
Select an output format	BAM
Don't output base quality	false
Write CIGAR with >65535 ops to the CG tag	false
Minibatch size for mapping (in megabyte)	Not available.
Output cs tag?	Nothing selected.
Generate CIGAR	false
Write =/X CIGAR operators	false
Use soft clipping for supplementary alignments ?	false

Table S2. Default parameters of the alignment of cleavage stage (10 hpf) reads against the *Otx* gene using BWA-MEM2.

**Input Parameter**	**Value**
Will you select a reference genome from your history or use a built-in index?	history
Use the following dataset as the reference	NM_001032368_genomic.fa
Single or Paired-end reads	paired_collection
Select a paired collection	SRR531949
Enter mean, standard deviation, max, and min for insert lengths.	Not available.
Set read groups information?	do_not_set
Select analysis mode	illumina
BAM sorting mode	Sort by chromosomal coordinates

Table S3. Default parameters of the alignment of cleavage stage (10 hpf) reads against the *Otx* gene using Bowtie2.

**Input Parameter**	**Value**
Is this single or paired library	paired_collection
FASTQ Paired Dataset	SRR531949
Write unaligned reads (in fastq format) to separate file(s)	false
Write aligned reads (in fastq format) to separate file(s)	false
Do you want to set paired-end options?	false
Will you select a reference genome from your history or use a built-in index?	history
Select reference genome	NM_001032368_genomic.fa
Set read groups information?	do_not_set
Select analysis mode	simple
Do you want to use presets?	No, just use defaults
Do you want to tweak SAM/BAM Options?	false
Save the bowtie2 mapping statistics to the history	false

Table S4. Default parameters of the alignment of cleavage stage (10 hpf) reads against the *Otx* gene using STAR.

**Input Parameter**	**Value**
Single-end or paired-end reads	paired_collection
RNA-Seq FASTQ/FASTA paired reads	SRR531949
Custom or built-in reference genome	history
Select a reference genome	NM_001032368_genomic.fa
Length of the SA pre-indexing string	14 (We did not find significant differences between coverage plots with default and other tested (10) conditions)
Build index with or without known splice junctions annotation	without-gtf
Per gene/transcript output	-
Diploid mode	false
Use 2-pass mapping for more sensitive novel splice junction discovery	None
twopass_read_subset	Empty.
sj_precalculated	Empty.
Report chimeric alignments?	Don't report chimeric alignments
oformat	
Read alignment tags to include in the BAM output	NH (number of reported alignments/hits for the read) HI (query hit index) AS (local alignment score) nM (number of mismatches per (paired) alignment) ch (used to indicate chimeric alignments)
HI tag values should be	one-based
outSAMprimaryFlag	OneBestScore
MAPQ value for unique mappers	60
Actiavte WASP filtering	
filter	
Exclude the following records from the BAM output	Nothing selected.

Input Parameter	Value
Would you like to set additional output filters?	false
algo	
Configure seed, alignment and limits options	default
perf	
Number of genome bins for coordinate-sorting	50
Maximum number of loci anchors are allowed to map to	50
Compute coverage	None
outWigStrand	false

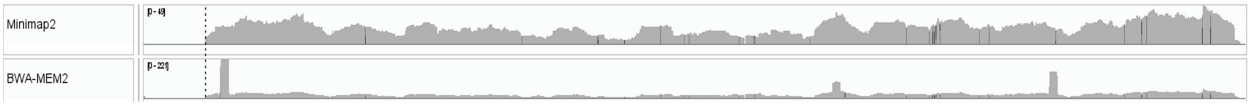

Figure S1. Coverage plot of the *Otx* gene third (last) exon of the by raw reads of the radial nerve. 5' end of the exon is marked by a dashed line.

Table S5. Expression analysis of six mRNA isoforms of the kinesin light chain protein gene (LOC373373) by single transcript mapping in radial nerve (SRR532046).

Expressed transcripts	Comments
XM_030998283	
XM_030998288	
XM_011676658	Impossible to check the 3'UTR length
Non-expressed transcripts	
XM_030998282	Coverage of the exon 12 3' end is absent, and exon 13 is not expressed
XM_030998279	Exon 13 is not expressed, reads almost non-overlap exon14-exon15 junctions
XM_030998284	Only a few reads overlap the exon13-exon14 junctions

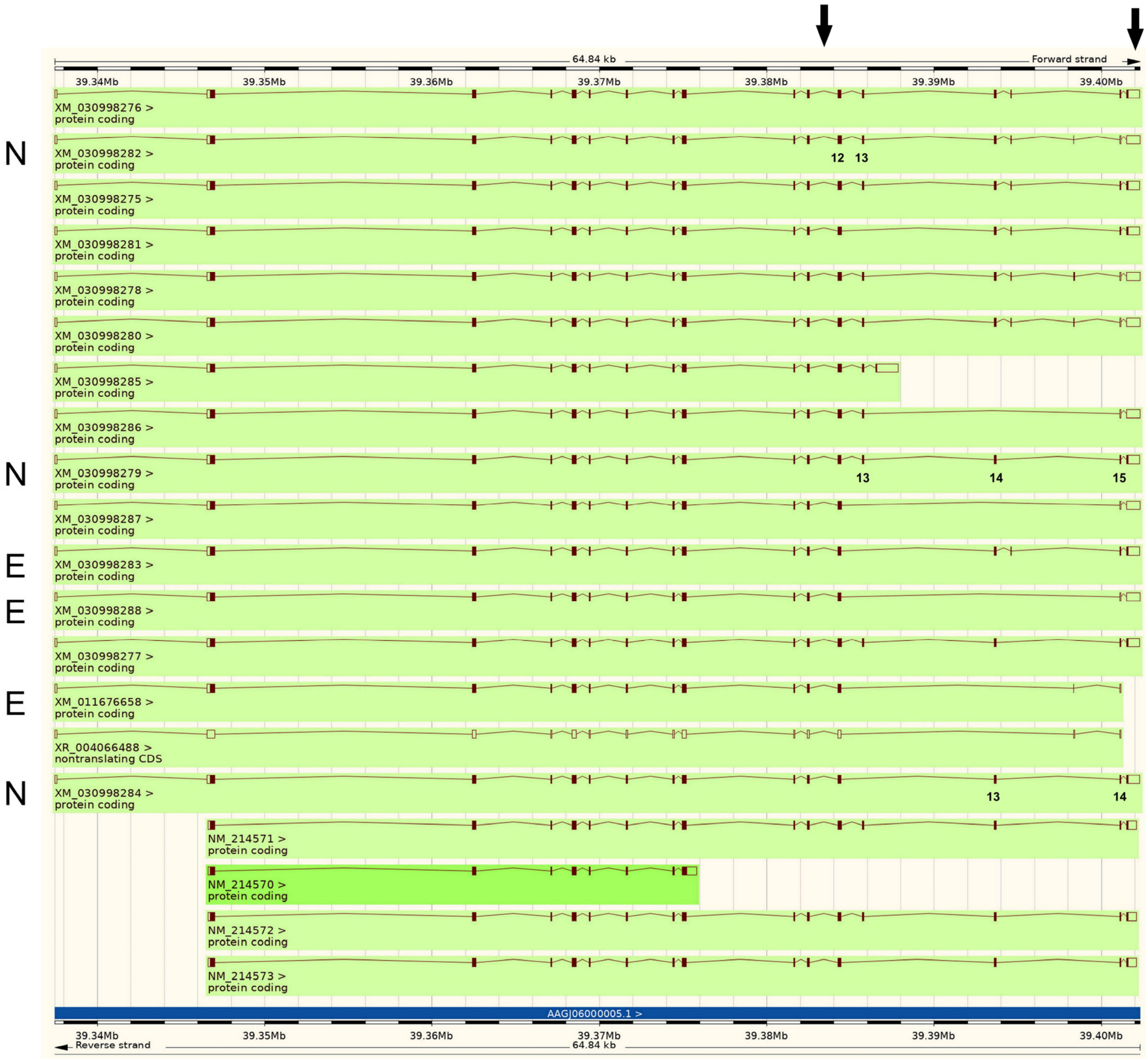

Figure S2. Structure of kinesin light chain gene (LOC373373). Analyzed isoforms are marked by letters. N – non-expressed isoforms, E – expressed isoforms. Region encoding alternatively spliced exons and alternative polyadenylation signals for the XM sequences is between the arrows. The number of exons that are mentioned in the next figures is listed below for each transcript sequence.

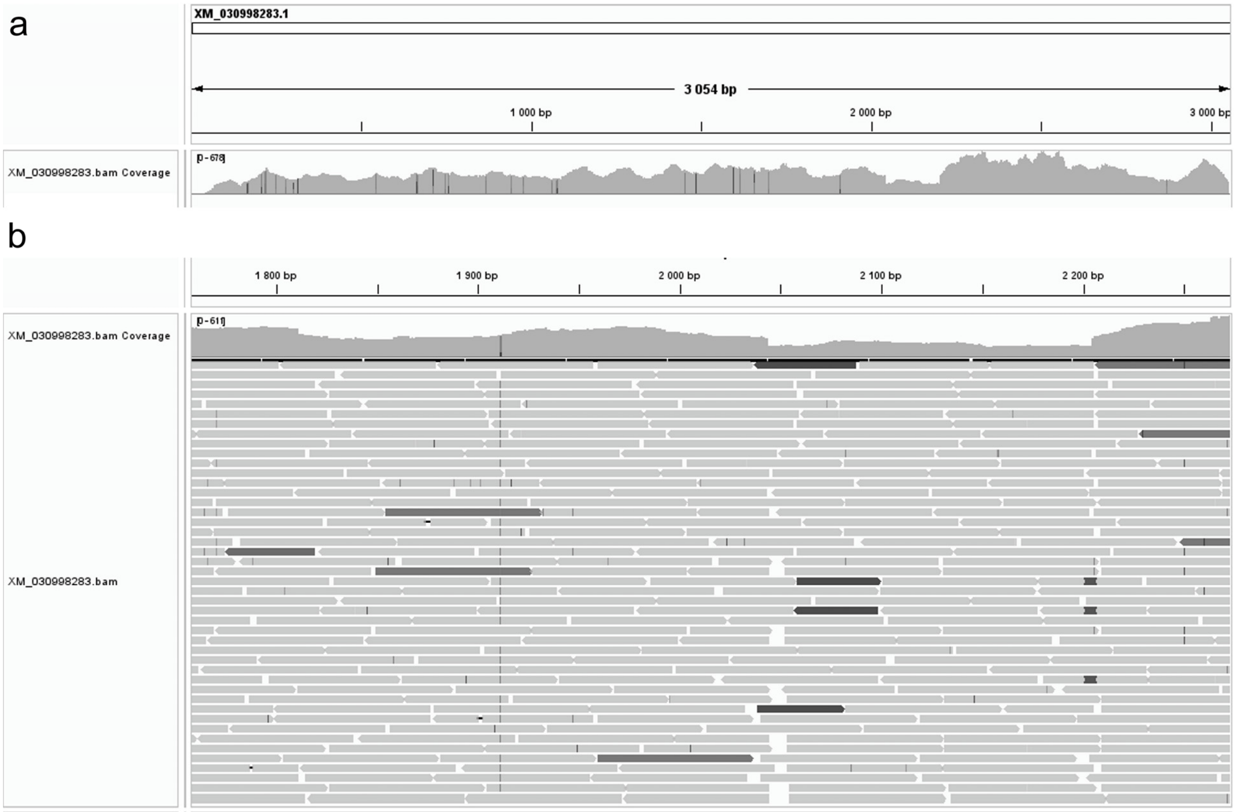

Figure S3. XM_030998283. transcript. (**a**) Coverage profile for the entire sequence. (**b**) Variable region.

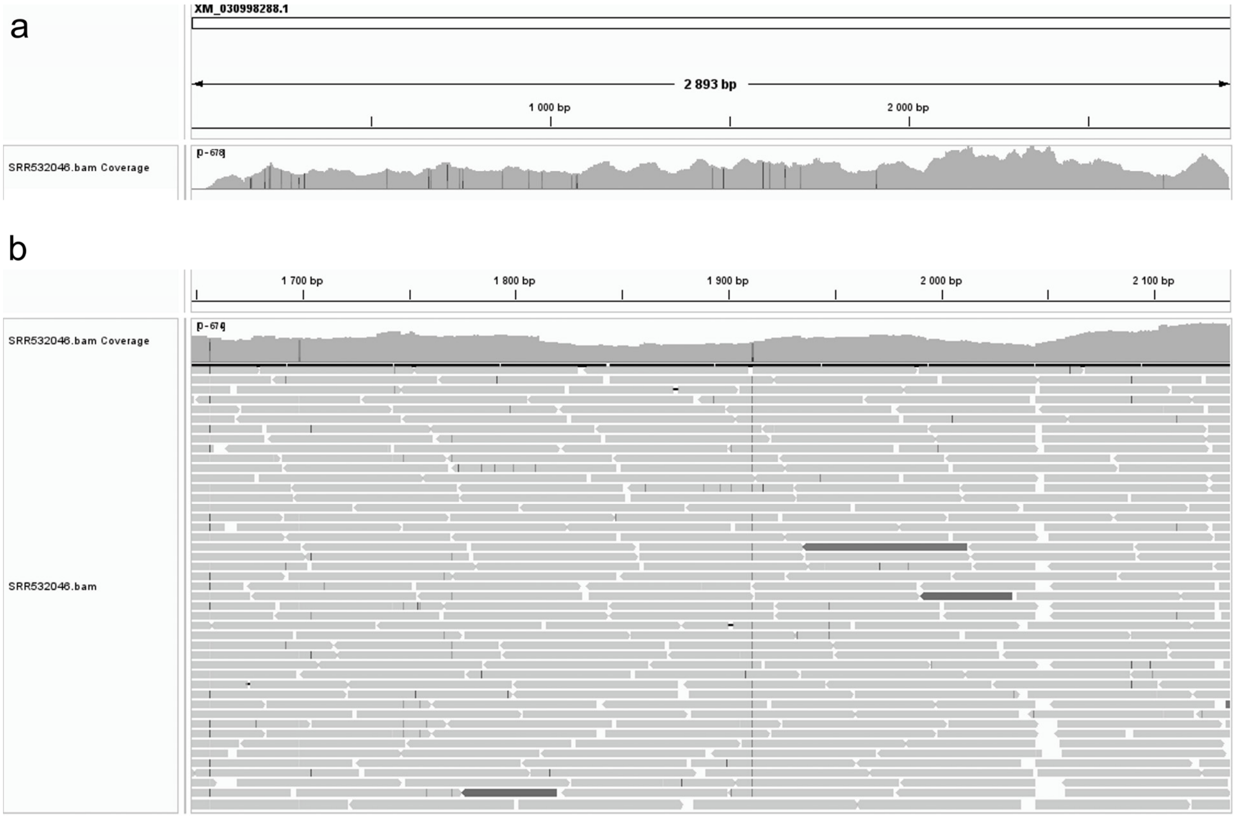

Figure S4. XM_030998288 transcript. (**a**) Coverage profile for the entire sequence. (**b**) Variable region.

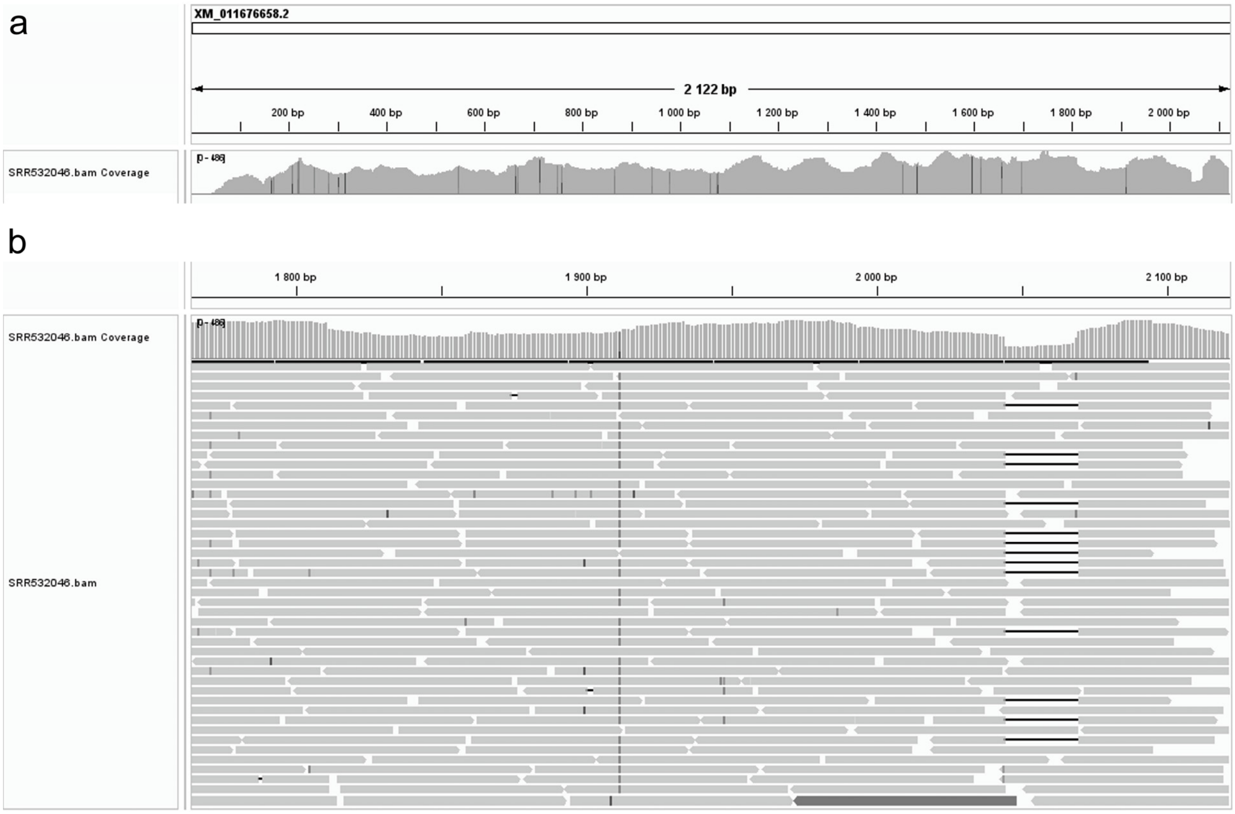

Figure S5. XM_011676658 transcript. (**a**) Coverage profile for the entire sequence. This transcript has shorter 3'UTR, and the coverage profile does not allow for checking this sequence by mapping. (**b**) Variable region. Gaps in read mapping (black lines) show alternative splicing of short exon.

Non-expressed isoforms

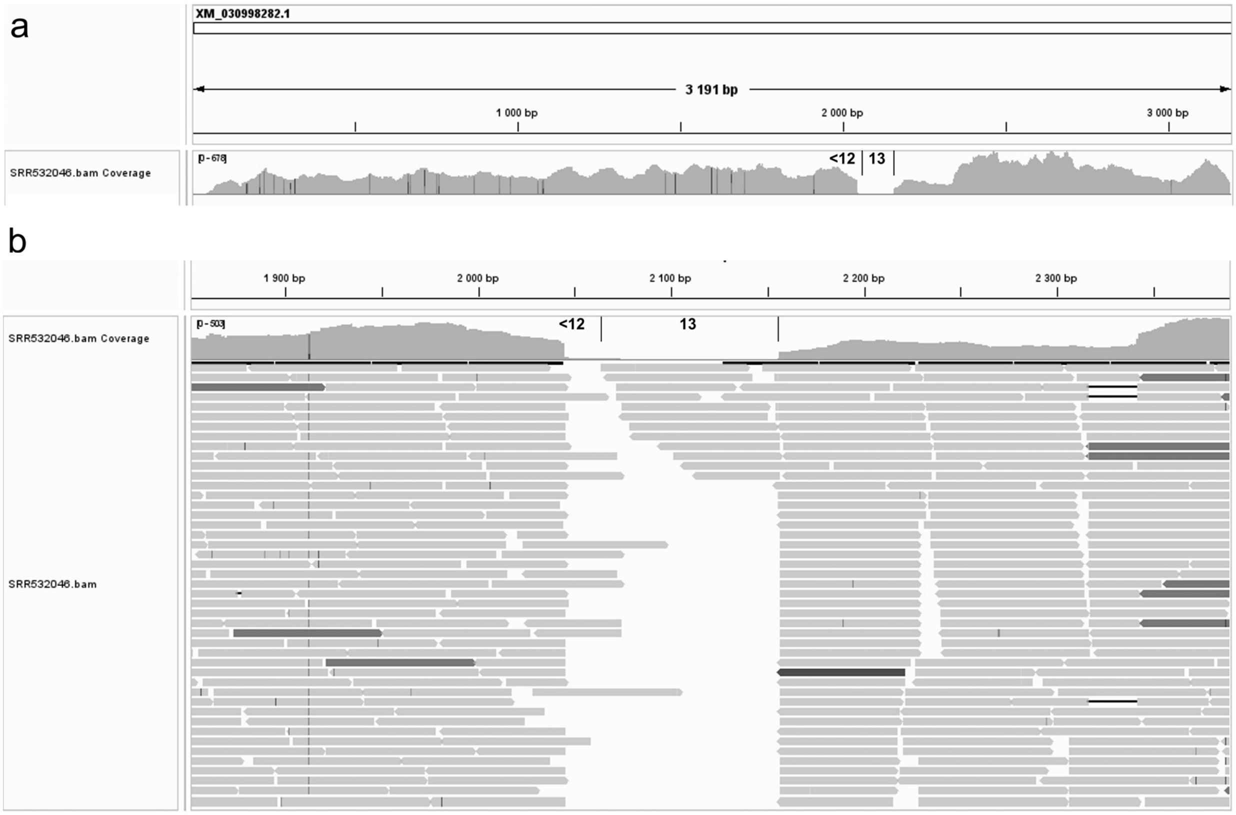

Figure S6. XM_030998282 transcript. (**a**) Coverage profile for the entire sequence. (**b**) Variable region. Coverage of the exon 12 3' end is absent. Therefore, exon 13 is not expressed. The ends of the exon 12 and exon 13 are marked in both images.

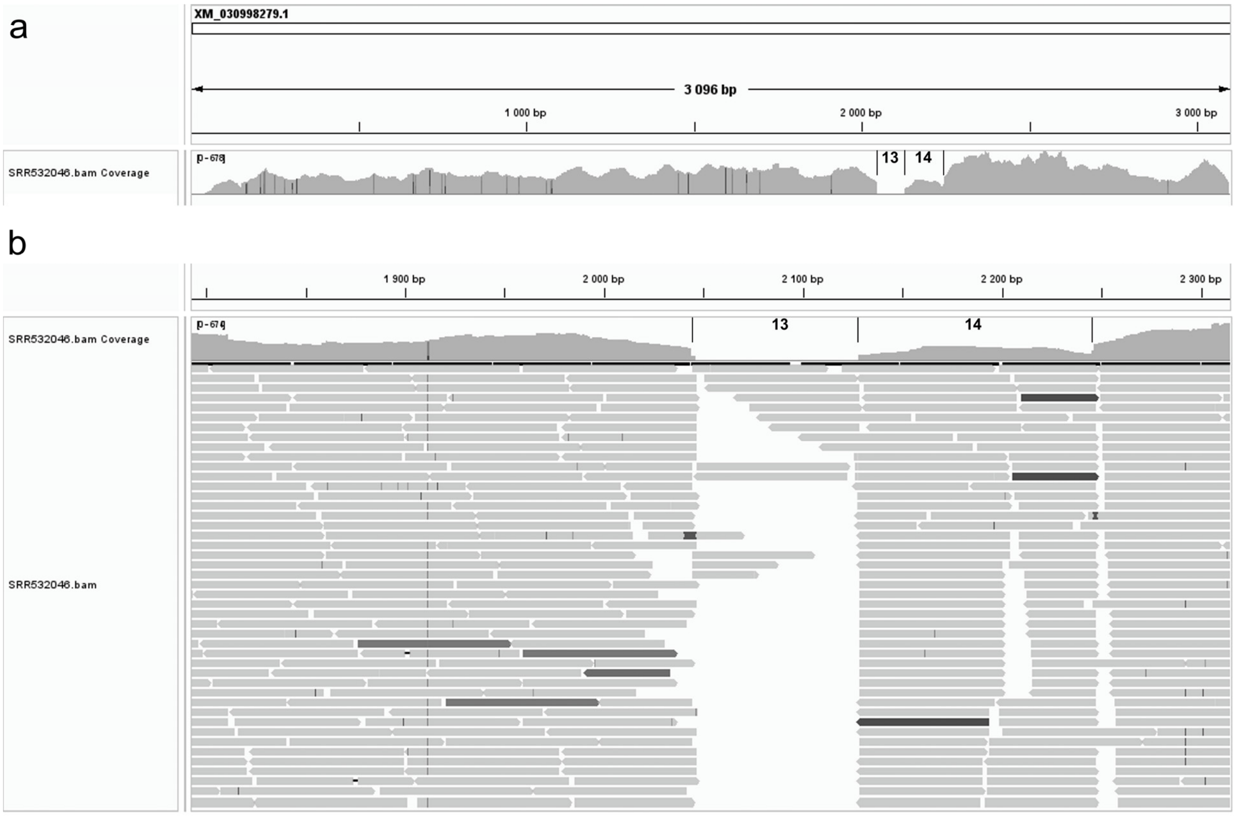

Figure S7. XM_030998279 transcript. (**a**) Coverage profile for the entire sequence. (**b**) Variable region. Exon 13 is not expressed, reads almost non-overlap exon14-exon15 junction. Exons 13 and 14 are marked in both images.

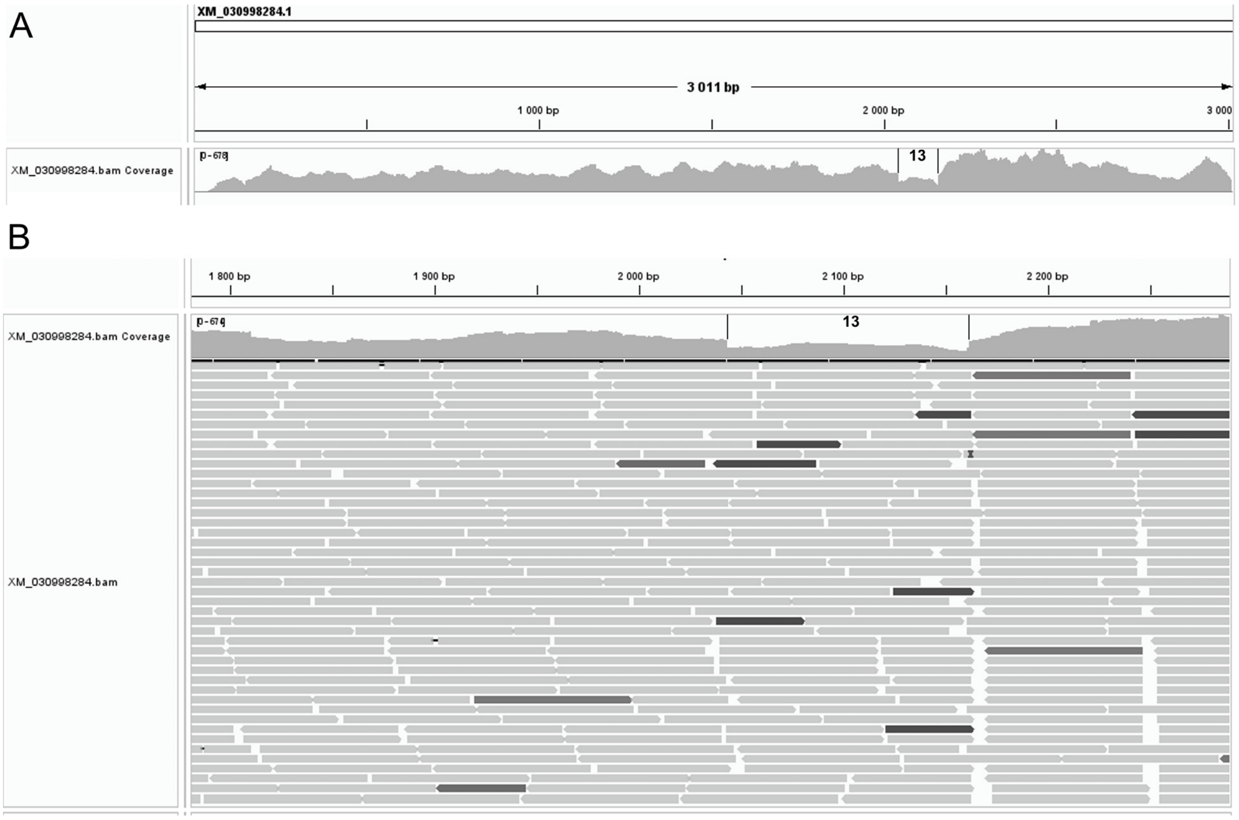

Figure S8. XM_030998284 transcript. (**a**) Coverage profile for the entire sequence. (**b**) Variable region. Reads almost non-overlap exon13-exon14 junction. Exon 13 is marked in both images.
